# Prophylactic Administration of Alpha Blocker for the Prevention of Urinary Retention in Males Undergoing Inguinal Hernia Repair Under Spinal Anesthesia: Interim Analysis of a Randomized Controlled Trial

**DOI:** 10.7759/cureus.19669

**Published:** 2021-11-17

**Authors:** Georgios D Koukoulis, Konstantinos Bouliaris, Konstantinos Perivoliotis, Konstantinos Tepetes

**Affiliations:** 1 School of Medicine, University of Thessaly, Larisa, GRC

**Keywords:** tamsulosin, alpha-blockers, urinary retention, spinal anesthesia, inguinal hernia repair

## Abstract

Introduction: This randomized controlled study aims to investigate the prophylactic effect of tamsulosin on the development of postoperative urinary retention (POUR) in men undergoing elective open inguinal hernia (IH) repair under spinal anesthesia. The study also focused on potentially predisposing factors for POUR.

Methods: 100 eligible patients were randomized into two groups. Patients in the experimental group were given two doses of tamsulosin 0.4 mg orally 24 hours and 6 hours before surgery. In the control group, two doses of placebo were administered, in the same manner as the study group. The following parameters were also recorded: the International Prostate Symptom Score (IPSS) questionnaire scores, the presence of scrotal hernia, operation duration, perioperative administration of IV opioids and/or atropine, postoperative pain, and preoperative anxiety.

Results: Overall, the incidence of POUR was 37% (37/100) with no difference between the two groups. Among patients receiving tamsulosin, 39.2% (20/51) developed POUR, compared to 34.7% (17/49) in the control group. Preoperative patients’ high anxiety visual analog scale (VAS) score (>51mm) (P=0.007) and the intraoperative use of atropine (P=0.02) were detected as risk factors for POUR.

Conclusion: This interim analysis of our prospective randomized trial showed no benefit from the prophylactic use of tamsulosin in preventing POUR after IH repair under spinal anesthesia. This type of anesthesia was also correlated with an overall high incidence of POUR. Preoperative anxiety and administration of atropine were identified as statistically significant factors for POUR. In patients with preoperative high anxiety, VAS score a different type of anesthesia may be used.

## Introduction

Inguinal hernia (IH) is the most common abdominal wall defect, with a lifetime risk of 27% for men and 3% for women. Surgical repair is the treatment of choice, and it is estimated that more than 20 million IH repairs are performed every year worldwide [1,2]. Moreover, a positive correlation between the IH incidence in men and age is noted, with the former being almost 200/10,000 person-years for patients aged 75 years and over [2].

Postoperative urinary retention (POUR) is a frequent adverse event after both emergency and elective procedures, with an incidence ranging from 5% to 70% [3]. POUR is generally defined as the postoperative inability to pass urine, but the definitions, still, vary widely. Regarding IH, POUR results in prolonged hospitalization and reduced patient satisfaction. Among the various risk factors that have been proposed are the male gender, elderly patients, history of benign prostatic hypertrophy (BPH), and spinal anesthesia [3]. The latter has been also confirmed in a previous study from our group, where 32% of patients under spinal anesthesia developed POUR [4]. However, despite these, spinal anesthesia, still, remains an attractive option for IH repair [5], since regional anesthesia is associated with favorable results in terms of hypotension, postoperative nausea, vomiting, and pain [3,6].

In order to reduce the incidence of POUR after IH repair, several authors have proposed the prophylactic use of alpha receptors’ antagonists [7-10]. However, there is, still, no consensus on whether prophylactic alpha-blockers administration can reduce rates of POUR in adult males.

The aim of this double-blinded, controlled randomized study was to investigate the prophylactic effect of tamsulosin, a selective alpha-1 adrenergic blocking agent, on the development of POUR in men undergoing elective open IH repair under spinal anesthesia.

## Materials and methods

The study was approved by the Hospital Ethics Committee and all participants provided written informed consent. The trial protocol was registered in ClinicalTrials.gov (NCT03976934).

Since September 2019, all male patients of 50 years and older, referred to the Outpatient Clinic of our Surgical Department for elective unilateral IH repair were evaluated for their eligibility. The following exclusion criteria were considered: 1) American Society of Anesthesiologists (ASA) score >3, 2) female patients, 3) history of orthostatic hypotension, 4) prostatic hypertrophy, 5) neurological diseases, 6) previous lower urinary tract operations, 7) complicated IHs, 8) administration of general or local anesthesia, and 9) patients with contraindication for tamsulosin administration.

All eligible patients were admitted one day prior to operation and were randomized into two groups. Patients in the experimental group were given two doses of tamsulosin 0.4 mg orally 24 hours and 6 hours before surgery. In the control group, two doses of placebo were administered, in the same manner as the study group. Randomization was based on a computer-generated table of random numbers. Opaque and sealed envelopes, numbered for each subject, were used and opened upon the arrival of the patient to the surgical clinic. All hernia repairs were done in a tension-free manner, with plug and/or mesh placement under spinal anesthesia. Postoperative management was standardized for all patients and included paracetamol 1 g every 8 hours, low molecular weight heparin and omeprazole. The patients were encouraged to mobilize. Per os feeding was administered, provided the absence of nausea and vomiting.

The primary endpoint of our study was the difference between the experimental and the control group in terms of POUR. POUR was defined as the inability to void 8 hours postoperatively. The following parameters were also recorded: the International Prostate Symptom Score (IPSS) questionnaire scores, the presence of scrotal hernia, operation duration, perioperative administration of IV opioids and/or atropine, postoperative pain, and preoperative anxiety. Pain assessment was based on the visual analog scale (VAS) score at 6, 12, and 24 hours after the operation (VAS score scale from 0 to 10, 0 no pain, 10 max pain). Preoperative anxiety was quantified by the anxiety VAS (A-VAS: 0-100 mm) score. A-VAS scores were summarized in two subgroups (Low A-VAS: 0-50 mm and high A-VAS: 51-100 mm), based on the respective literature reports [11].

Statistical analysis

Prior to any statistical analyses, all data underwent a Shapiro-Wilk normality test. In variables where normality was confirmed, a parametric approach was applied; in any other cases, a non-parametric analysis was implemented. Independent samples’ t-test and Mann-Whitney U test were used for the comparison of normal and non-normal continuous variables, respectively. Pearson chi-square test was calculated for categorical variables. The relation between two continuous data was assessed with a regression analysis. To further confirm the factors associated with the abovementioned study outcomes, a logistic regression model was used. The effect estimates of these analyses were displayed with the corresponding odds ratio (OR) and 95% CIs. Based on the normality test results, continuous data was reported as mean (standard deviation) or median (interquartile range-IQR). Moreover, categorical variables were reported as N (percentage). Statistical significance was considered at the level of P<0.05. All analyses were performed in SPSS Statistics v.22 software (SPSS Inc. Chicago, IL, USA). Sample size analysis indicated a total sample size of 196 patients (98 per group) to detect a 50% decrease in the POUR rate (32%) when a1 blocker was administered. An interim analysis was planned after completion of the first half of patients and the results are presented and discussed herein.

## Results

Between September 2019 and June 2021, 100 patients were randomized to either the tamsulosin group (group 1, n: 51) or the control group (group 2, n: 49). The mean age was 63.54 years. In total, 73 indirect, 25 direct, and two combined hernias were included. No statistically significant differences in terms of base demographics were found (Table 1). In 75 patients, a mesh and plug combination was introduced, whereas a mesh or a plug-only approach was applied in 18 and seven patients, respectively. Operation duration was comparable between the two groups. Overall, the incidence of POUR was 37% (37/100) with no difference between the two groups. Among patients receiving tamsulosin, 39.2% (20/51) developed POUR compared to 34.7% (17/49) in the control group. Overall, eight patients had IPSS scores >15. Bladder catheterization was applied in all POUR cases according to the study’s protocol followed by an attempt for removal the next morning. Catheter removal was successful in less than 24 hours in 34 patients (17 patients in each group), while in one patient the catheter was removed on the second postoperative day. Two patients required prolonged catheterization.

**Table 1:** Patient demographics and perioperative characteristics of inguinal hernia repairs *normality confirmed. Normal values presented as mean (SD). t-test applied. Non-normal values presented as median (IQR). Mann-Whitney U test applied. EHS class:  European Hernia Society Classification; IPSS: International Prostate Symptom Score; IQR: interquartile range; VAS: visual analog scale

			A Blocker		Total	P value
			Yes (51)	No (49)		
Age*			64.76 (10.25)	62.27 (10.19)	63.54 (10.25)	0.226
Previous Abdominal Operations			10 (19.6%)	13 (26.5%)	23 (23.0%)	0.411
IPSS		Mild (0-7)	35 (68.6%)	31 (63.3%)	66 (66.0%)	0.662
		Moderate (8-19)	14 (27.5%)	17 (34.7%)	31 (31.0%)	
		Severe (20-35)	2 (3.9%)	1 (2.0%)	3 (3.0%)	
Weight (kg)			78 (13)	77 (9)	77 (13)	0.325
Height* (m)			1.72 (0.07)	1.71 (0.07)	1.72 (0.07)	0.563
BMI			25.7 (4.1)	25.3 (3.8)	25.6 (4.1)	0.526
A-VAS Score		Low (0-50 mm)	36 (70.6%)	41 (83.7%)	77 (77.0%)	0.12
		High (51-100 mm)	15 (29.4%)	8 (16.3%)	23 (23.0%)	
Hernia Type	Indirect		36 (70.6%)	37 (75.5%)	73 (73.0%)	0.365
	Direct		13 (25.5%)	12 (24.5%)	25 (25.0%)	
	Combined		2 (3.9%)	0 (0.0%)	2 (2.0%)	
Mesh	Combined		37 (72.5%)	38 (77.6%)	75 (75.0%)	0.605
	Mesh		11 (21.6%)	7 (14.3%)	18 (18.0%)	
	Plug		3 (5.9%)	4 (8.2%)	7 (7.0%)	
EHS Class	L1		14 (27.5%)	15 (30.6%)	29 (29.0%)	0.252
	L2		13 (25.5%)	18 (36.7%)	31 (31.0%)	
	L3		11 (21.6%)	4 (8.2%)	15 (15.0%)	
	M1		2 (3.9%)	3 (6.1%)	5 (5.0%)	
	M2		5 (9.8%)	7 (14.3%)	12 (12.0%)	
	M3		6 (11.8%)	2 (4.1%)	8 (8.0%)	
Scrotal Hernia			9 (17.6%)	7 (14.3%)	16 (16.0%)	0.647
Hernia Sac Size	<10 cm		36 (70.6%)	39 (79.6%)	75 (75.0%)	0.299
	>10 cm		15 (29.4%)	10 (20.4%)	25 (25.0%)	
Operation Duration (min)			46 (21)	42 (25)	45 (25)	0.205
VAS 6h			2 (2)	2 (2)	2 (2)	0.919
VAS 12h			2 (1)	2.58 (2)	2 (2)	0.661
VAS 24h			2 (1)	2 (2)	2 (1)	0.413
Intraoperative Fluids (L)			1 (1)	1 (0.75)	1 (1)	0.352
Postoperative Fluids (L)			0.5(0)	0.5 (0)	0.5 (0)	0.755
Total Fluids (L)			2 (1)	1.5 (1)	1.5 (1)	0.316
Spinal Opioids			22 (43.1%)	24 (49.0%)	46 (46.0%)	0.558
IV Opioids			14 (27.5%)	22 (44.9%)	36 (36.0%)	0.06
Atropine			2 (3.9%)	6 (12.2%)	8 (8.0%)	0.125
Need for extra Postoperative Analgesia			4 (7.8%)	2 (4.1%)	6 (6.0%)	0.428
Urinary Retention			20 (39.2%)	17 (34.7%)	37 (37.0%)	0.64

Preoperative patients’ high anxiety VAS score (>51mm) (P=0.007) and the intraoperative use of atropine (P=0.02) were detected as risk factors for POUR (Table 2). Regression analysis confirmed the results (Table 3).

**Table 2:** Patient, surgical, and perioperative risk factors for POUR *normality confirmed. Normal values presented as mean (SD). t-test applied Non-normal values presented as median (IQR). Mann-Whitney U test applied. EHS class:  European Hernia Society Classification; IPSS: International Prostate Symptom Score; IQR: interquartile range; VAS: visual analog scale

		Urinary Retention		Total	P value
		Yes (37)	No (63)		
Age*		63.96 (9.7)	63.29 (10.6)	63.5 (10.2)	0.755
Previous Abdominal Operations		6 (16.2%)	17 (27.0%)	23 (23.0%)	0.217
IPSS	Mild (0-7)	20 (54.1%)	46 (73.0%)	66 (66.0%)	0.127
	Moderate (8-19)	16 (43.2%)	15 (23.8%)	31 (31.0%)	
	Severe (20-35)	1 (2.7%)	2 (3.2%)	3 (3.0%)	
Weight (kg)		79 (12)	76 (12)	77 (13)	0.791
Height* (m)		1.72 (0.07)	1.71 (0.07)	1.72 (0.07)	0.451
BMI		25.5 (4.75)	25.8 (4.3)	25.6 (4.1)	0.214
A-VAS Score	Low (0-50 mm)	23 (62.2%)	54 (85.7%)	77 (77.0%)	0.007
	High (51-100 mm)	14 (37.8%)	9 (14.3%)	23 (23.0%)	
Hernia Type	Indirect	25 (67.6%)	48 (76.2%)	73 (73.0%)	0.635
	Direct	11 (29.7%)	14 (22.2%)	25 (25.0%)	
	Combined	1 (2.7%)	1 (1.6%)	2 (2.0%)	
Mesh	Combined	23 (62.2%)	52 (82.5%)	75 (75.0%)	0.053
	Mesh	11 (29.7%)	7 (11.1%)	18 (18.0%)	
	Plug	3 (8.1%)	4 (6.3%)	7 (7.0%)	
EHS Class	L1	12 (32.4%)	17 (27.0%)	29 (29.0%)	0.617
	L2	10 (27.0%)	21 (33.3%)	31 (31.0%)	
	L3	4 (10.8%)	11 (17.5%)	15 (15.0%)	
	M1	2 (5.4%)	3 (4.8%)	5 (5.0%)	
	M2	4 (10.8%)	8 (12.7%)	12 (12.0%)	
	M3	5 (13.5%)	3 (4.8%)	8 (8.0%)	
Scrotal Hernia		3 (8.1%)	13 (20.6%)	16 (16.0%)	0.09
Hernia Sac Size	<10 cm	31 (83.8%)	44 (69.8%)	75 (75.0%)	0.12
	>10 cm	6 16.(2%)	19 (30.2%)	25 (25.0%)	
Operation Duration (min)		46 (26)	45 (22)	45 (25)	0.327
VAS 6h		2 (3)	2 (1)	2 (2)	0.1
VAS 12h		2 (1)	2 (1)	2 (2)	0.811
VAS 24h		2 (1)	2 (2)	2 (1)	0.779
Intraoperative Fluids (L)		1 (1)	1 (1)	1 (1)	0.859
Postoperative Fluids (L)		0.5 (0)	0.5 (0)	0.5 (0)	0.109
Total Fluids (L)		1.5 (1)	1.5 (1)	1.5 (1)	0.609
Spinal Opioids		19 (51.4%)	27 (42.9%)	46 (46.0%)	0.411
IV Opioids		9 (24.3%)	27 (42.9%)	36 (36.0%)	0.06
Atropine		6 (16.2%)	2 (3.2%)	8 (8.0%)	0.02
Need for extra Postoperative Analgesia		3 (8.1%)	3 (4.8%)	6 (6.0%)	0.496
A-Blocker		20 (54.1%)	31 (49.2%)	51 (51.0%)	0.64

**Table 3:** Logistic regression model OR: odds ratio

Factors	OR 95% CI	P value
Atropine	6.85 (1.18-39.8)	0.032
A-VAS (high 50-100 mm)	3.29 (1.02-10.62)	0.046

No complications or side effects of therapy were encountered during the treatment with tamsulosin or placebo.

## Discussion

Urinary retention is a common complication after any surgical procedure and especially after IH repair [12]. Although POUR is considered a minor complication, it is painful and often requires catheterization for relief which can cause urethral trauma or catheter-related infections, it delays discharge and increases costs [13].

POUR in male patients undergoing IH repair varies widely in published series ranging from less than 1% to greater than 34% which can be attributed to many factors [14]. The underlying physiological mechanism leading to POUR relates to α-adrenergic overstimulation following IH repair. Sympathetic nerve activity during the perioperative period leads to catecholamine release and α-adrenergic stimulation of bladder neck muscles preventing bladder emptying [14]. The innervation of the lower urinary tract is mainly by sympathetic and parasympathetic divisions of the autonomic nervous system and somatic portions of the pudendal nerve. The parasympathetic efferent nerves via various muscarinic receptors in bladder smooth muscles excite the bladder and relax the urethra while the sympathetic efferent nerves inhibit the bladder body and excite the bladder base and urethra nerve. The smooth muscle of the bladder is also rich in β-receptors which initiate relaxation when stimulated by norepinephrine or epinephrine while the bladder neck and urethra contain mainly α-receptors that initiate contraction when stimulated by norepinephrine [15].

Suggested factors which may interrupt with the voiding reflex after IH are the perioperative fluid management, the type of anesthesia, the use of narcotic analgesia, increased outlet resistance, postoperative pain, and patient age and sex [12]. Excessive perioperative fluid intake can lead to bladder overdistention which increases the risk of POUR [12]. The type of anesthesia can also affect the incidence of POUR, particularly when general or regional anesthesia is used. General anesthetics may cause bladder atony while regional anesthesia may interrupt the micturition reflex leading to detrusor blockage [13]. On the other hand, outlet closure is done via increasing α-receptors-mediated tone in the bladder outlet [16]. Certain sympathomimetic and anticholinergic drugs, such as phenylephrine and atropine, inhibit bladder tone during surgery leading to a distended bladder with decreased urge to void. In the postoperative period, pain in the groin area can also stimulate α-adrenoreceptors in the prostate and proximal urethra causing increased urethral and bladder resistance which can lead to retention [17]. Furthermore, it is generally accepted that the incidence of POUR increases in males with age, and one of the most likely causes of this is BPH. One hypothesis for the cause of urinary retention occurring in the presence of BPH following IH repair is due to adrenergic overstimulation of the smooth muscle in the bladder neck and prostate which are rich in α-adrenergic receptors [3].

The rationale for pharmacologic prevention of POUR is based on increasing detrusor contractility or relaxing proximal urethra. Alpha-adrenergic blockers act by reducing the tone in the bladder outlet and thus decrease outflow resistance and facilitate micturition. Prophylactic administration of these drugs has been shown to be effective in preventing POUR after IH repair, and in the latest international guidelines for groin hernia management, there is a statement that prazosin, phenoxybenzamine hydrochloride, or tamsulosin may be effective in preventing urinary retention [18]. In six published prospective studies comparing POUR rates after elective unilateral IH repair, 625 patients received prophylactic alpha-blockers vs placebo or no treatment. All studies included males only and four of the six studies included only those over the age of 50 [7-9,19], while one study included males between the ages of 20 and 70 [10] and one study included males 18 years of age or older [20]. The prophylactic alpha-blocker was tamsulosin in three studies [7,8,20], prazosin in two studies [10,19], and phenoxy benzamine in one study [9]. Treatment regimens varied in time of dosage while in one study, no placebo was used in the control group [9]. The type of anesthesia was either spinal or general anesthesia [7,9,19], general anesthesia only [10,20], or not specified [8]. The method of IH repair was described only in two studies as open [7,10]. In five studies, group comparability was ensured by assessing preoperative urinary function with a number of internationally recognized assessment scores and tools [7-10,19]. In four studies, there was a statistically significant reduction in POUR rates in the groups receiving alpha-blocker compared to placebo [7-10] while two studies found no improvement in retention rates [19-20].

In the present study, we investigated the prophylactic effect of tamsulosin, a selective alpha-1a adrenergic blocking agent, on the development of POUR in men ≥ 50 years old undergoing elective open IH repair. Tamsulosin was chosen on the basis that is inexpensive, easy to administer, has a low adverse effect, profile and reaches peak serum levels at 4 hours after administration. Since there is limited data in the literature regarding the timing of tamsulosin administration in preventing POUR after IH repair, we decided to administrate the drug 24 hours and 6 hours before surgery, similar to the Mohammadi-Fallah et al. study [7]. This way was practical, ensured two doses of tamsulosin before surgery with a high interval time between the doses, and allowed patients’ monitoring for the development of any adverse events before surgery.

Our results showed no difference in the rates of POUR between the tamsulosin group and the control group which is not in accordance with the results of other studies which used the same drug [7,8]. Possible reasons for this could be that we focused only on patients receiving spinal anesthesia, the time frame for development of urinary retention was shorter (8 hours), and we used different times of tamsulosin dosage than in the other two studies. On the other hand, Caparelli et al. also used tamsulosin in their study and similar to us found no improvement in POUR rates between the placebo group and the tamsulosin group. However, in their study, only patients with laparoscopic IH repair were included [20].

The short time frame for the diagnosis of POUR (only 8 hours) in our study can also explain the overall high incidence of urinary retention (37%). Another reason could be the use of spinal anesthesia which predisposes to higher rates of POUR after IH repair [4,13] and the fact that in near half of the patients (46%) opioids were used for the spinal anesthesia [21].

Regarding the predisposing factors of POUR, only preoperative anxiety related to the surgical procedure and the intraoperative use of atropine were statistically significant. The importance of the A-VAS score is that it can be easily measured and can the patients with higher risk for POUR. These patients might need a different approach like a thorough explanation of their surgery or an alternative type of anesthesia.

Our study holds limitations. This is an interim analysis of a single-center study, with a small number of patients included. We also used VAS scores for assessing preoperative anxiety and postoperative pain, even though A-VAS and P-VAS have high sensitivity and specificity, these are subjective methods.

## Conclusions

In conclusion, this interim analysis of our prospective randomized trial showed no benefit from the prophylactic use of tamsulosin in preventing POUR after IH repair under spinal anesthesia. This type of anesthesia was also correlated with an overall high incidence of POUR.

The study also focused on potentially predisposing factors for POUR. Among the measured factors, only preoperative anxiety and the intraoperative use of atropine were identified as statistically significant factors. In patients with preoperative high anxiety, VAS score of a different type of anesthesia may be used.
